# A NOVEL MRI-COMPATIBLE SYSTEM CHARACTERIZING THE EFFECTS OF AGING ON SUPRASPINAL SENSORIMOTOR CONTROL OF WALKING

**DOI:** 10.1093/geroni/igae098.2232

**Published:** 2024-12-31

**Authors:** Hao Yue, Bin Shen, Yishu Chen, Yufeng Zhang, Jiaojiao Lü, Brad Manor, WeiJie Fu, Junhong Zhou

**Affiliations:** Guizhou University, Guiyang, Guizhou, China (People’s Republic); Shanghai University of Sport, Shanghai, Shanghai, China (People’s Republic); Shanghai University of Sport, Shanghai, Shanghai, China (People’s Republic); Shanghai University of Sport, Shanghai, Shanghai, China (People’s Republic); Shanghai University of Sport, Shanghai, Shanghai, China (People’s Republic); Harvard Medical School/Hebrew SeniorLife, Boston, Massachusetts, United States; Shanghai University of Sport, Shanghai, Shanghai, China (People’s Republic); Harvard Medical School/Hebrew SeniorLife, Boston, Massachusetts, United States

## Abstract

As the only part in direct contact with the ground during walking, foot soles continuously perceive the somatosensory information (e.g., ground reaction forces). The activation of the supraspinal regions/networks in response to such somatosensory inputs is thus important for walking performance, which is oftentimes altered by aging and age-related conditions. It is challenging to characterize such supraspinal activations via traditional neuroimaging techniques (e.g., functional MRI, fMRI), since people are required to stay motionless during the MRI scan. We here thus developed a novel foot-sole stimulation system that simulates the pressure changes as experienced by each foot sole and the pace of foot switch during walking over the ground, and enables characterizing the walking-related activation of the supraspinal regions via fMRI. To examine its validity and reliability of simulation, 10 younger and 10 older adults completed two trials of 10-meter walking. The recorded temporal changes of pressure on foot soles of each step were recorded and used to program the motion of the system with high validity and reliability (r=0.90~0.95, p< 0.0001). The phantom imaging test showed that the signal-to-noise ratio of the MR image in system-working (29.84±4.32) was similar to that (29.78±3.76) in off-working condition (p=0.73), suggesting great MRI compatibility. The block-designed fMRI test showed that compared to resting, multiple supraspinal regions (e.g., postcentral and precuneus gyrus) (p< 0.005) were activated by the foot-sole stimulation. This novel MRI-compatible system provides a novel tool to characterize the effects of aging and age-related conditions on the supraspinal sensorimotor control of walking.

